# Diversified Phenomena in Metal- and Transition-Metal-Adsorbed Graphene Nanoribbons

**DOI:** 10.3390/nano11030630

**Published:** 2021-03-03

**Authors:** Shih-Yang Lin, Ngoc Thanh Thuy Tran, Ming-Fa Lin

**Affiliations:** 1 Department of Physics, National Chung Cheng University, Chiayi 621301, Taiwan; sylin.1985@gmail.com; 2 Hierarchical Green-Energy Materials (Hi-GEM) Research Center, National Cheng Kung University, Tainan 70101, Taiwan; 3 Department of Physics, National Cheng Kung University, Tainan 70101, Taiwan; mflin@mail.ncku.edu.tw; 4 Quantum Topological Center, National Cheng Kung University, Tainan 70101, Taiwan

**Keywords:** graphene nanoribbon, first-principles, adsorption, electronic, magnetic

## Abstract

Adatom-adsorbed graphene nanoribbons (GNRs) have gained much attention owing to the tunable electronic and magnetic properties. The metal (Bi, Al)/transition metal (Ti, Fe, Co, Ni) atoms could provide various outermost orbitals for the multi-orbital hybridizations with the out-of-plane π bondings on the carbon honeycomb lattice, which dominate the fundamental properties of chemisorption systems. In this study, the significant similarities and differences among Bi-/Al-/Ti-/Fe-/Co-/Ni-adsorbed GNRs are thoroughly investigated by using the first-principles calculations. The main characterizations include the adsorption sites, bond lengths, stability, band structures, charge density distributions, spin- and orbital-projected density of states, and magnetic configurations. Furthermore, there exists a transformation from finite gap semiconducting to metallic behaviors, accompanied by the nonmagnetism, antiferromagnetism, or ferromagnetism. They arise from the cooperative or competitive relations among the significant chemical bonds, finite-size quantum confinement, edge structure, and spin-dependent many-body effects. The proposed theoretical framework could be further improved and generalized to explore other emergent 1D and 2D materials.

## 1. Introduction

Easy synthesis, able to open band gaps, and other remarkable properties have inspired a host of studies on graphene nanoribbons (GNRs), which is a one-dimensional (1D) narrow strip of graphene [1,2,3]. In addition to the honeycomb lattice with a very active chemical environment, the 1D quantum confinement effects in the presence of the achiral and chiral edge structures of GNRs can greater diversify the essential properties compared to 2D graphene. Typically, there are two common types of GNRs, the armchair and zigzag ones (AGNRs and ZGNRs) [4,5], as classified by the ribbon edge’s structure. The former belong to nonmagnetic (NM) semiconductors, while the latter are antiferromagnetic (AFM) middle-gap semiconductors. On the other hand, the nanoribbon width also plays critical roles in the essential properties of GNRs. It is predicted to be inversely proportional to the energy gap, indicating the quantum-confinement effect. This width-dependent bandgaps of GNRs can be identified by scanning tunneling spectroscopy (STS) measurement [6]. Up to now, GNRs have been successfully synthesized by various experimental methods under the top-down and bottom-up schemes, e.g., via lithographic [6,7], sonochemical breaking [8], oxidization reaction [9], chemical vapor deposition [10], unzipping CNTs using plasma etching [11], etc. Recently, GNRs have become promising in the fields of the semiconductor industry and energy storage, for instance field-effect transistors [8,12], Li-ion batteries (LIBs) [13,14], and fuel cells [15,16]. GNRs with a width below 10 nm were found to be semiconductors and can serve as field effect transistors at room temperature with on-off ratios of about 10${}^{7}$ [7,16]. Apart from this, the sub-10 nm GNR showed a superior capacity of 490.4 mAh/g after 100 cycles [14], much higher compared to that of graphite, the commercialized anode of LIBs. To further expand the range of application, GNRs’ properties can be modulated by changing the geometric structures [17,18], doping [19,20], and applying external electric/magnetic fields [21,22].

Chemical modification on GNRs is the most effective strategy in creating dramatic changes. The adatom-adsorbed GNRs have been the focus of a number of theoretical [23,24,25] and experimental [26,27] studies. This work will focus on two types of adatoms: metals (Al, Bi) and transition metals (Ti, Fe, Co, Ni). After adsorption on GNR surfaces, they are expected to induce more complicated multi-orbital hybridizations in the significant bonds with carbon atoms, especially the 2p${}_{z}$ orbital. These systems are very suitable for exploring the dramatic transformations induced by adsorption, e.g., the semiconductor-metal transition and the diverse spin distributions. For metal-doped systems, they are predicted to have very high conduction electron densities and thus high electrical conductivities, which is promising for anode materials in rechargeable batteries. Aluminum atoms have been identified to play a critical role in the great enhancement of current density in an Al-ion battery [28], where the predominant AlCl${}_{4}$ anions are intercalated and de-intercalated between graphite layers during charge and discharge, respectively. On the other hand, bismuth oxide on nickel foam covered with thin carbon layers showed good performance as an anode of LIBs [29]. As for transition metal adatom (Ti, Fe, Co, Ni) doped systems, they are predicted to induce metallic band structures with free conduction electrons, in which the spin-split energy bands correspond to the ferromagnetic configuration [30,31]. However, the geometrical relationships among the structures, the electronic properties, and the magnetic configuration for various adatom coverages have not been thoroughly reported for GNRs. Orbital hybridizations between adatoms and GNRs, a key point in understanding the modification of essential properties, still lack a systematic investigation. Here, metals (Al, Bi) and transition metals (Ti, Fe, Co, Ni)-adsorbed GNRs will be able to create various orbital hybridizations and diversify the fundamental properties.

In this study, a theoretical framework by means of first-principles calculations is used to systematically investigate the diverse charge- and spin-created phenomena. A fitting question that might be asked is to which kinds of electronic properties (finite-gap/zero-gap semiconductors, semimetals, or metals) and magnetic configurations (ferromagnetism (FM), AFM, or NM) the material belongs. The answer lies in the way we deal with the delicate orbital hybridizations and spin configuration analysis. It is noteworthy that the former can be revealed from the optimal lattice symmetries, the atom-dominated energy bands, the atom- and orbital-projected densities of state (DOSs), and the spatial charge distributions. They could be used to identify the complex orbital hybridizations among different atom types of the studied material. Such chemical bonds play a critical role in the fundamental properties, accounting for the rich and unique geometric structures and electronic properties. Apart from the orbital hybridizations, the spin configuration is important for creating diverse magnetic configurations. By utilizing the polarized calculations, the magnetic configurations can be examined by using spin-split band structures, net magnetic moments, spin-density distribution, and spin-decomposed DOSs. This developed theoretical framework will be useful for characterizing not only GNRs, but also other 2D and 3D materials.

This work aims to investigate the geometric, electronic, and magnetic properties of metal/transition metal-adsorbed GNR systems. The essential properties arising from various types and concentrations of adatom dopings are studied in great detail. The calculations focus on the adatom-dependent binding energies, the adatom-carbon lengths, the optimal position, the maximum adatom concentrations, the adatom-related valence and conduction bands, the various van Hove singularities in DOSs, the adatom-induced magnetic properties, and the significant competitions of the carbon ribbon’s edge and the metal/transition metal adatoms in spin configurations. Moreover, distinct chemical bondings will be clearly identified under the delicate physical quantities.

## 2. Computational Methods

Our first-principles calculations were performed with the use of the density functional theory through the Vienna ab initio simulation software package (VASP) [32,33]. The projector augmented wave method was employed to evaluate the electron-ion interactions [34], whereas the electron-electron Coulomb interactions belong to the many-particle exchange and correlation energies under the Perdew–Burke–Ernzerhof generalized gradient approximation method [35]. To carefully explore the chemical adsorption effects on the magnetic properties, the spin configurations are taken into account. The 1D periodic boundary condition is along the x-axis, and the vacuum distance along the y- and z-axis is set equal to 15 in order to suppress the interactions between neighboring cells. A plane-wave basis set, with a maximum energy cutoff of 500 eV, is available in the calculations of Bloch wave functions. All atomic coordinates are relaxed until the Hellmann–Feynman force on each atom is less than 0.01 eV/ with energy convergence of 10${}^{-5}$ eV between two simulation steps. The pristine first Brillouin zone is sampled in a Gamma scheme along the 1D periodic direction by 30×1×1 k-points for structure relaxations and then by 100×1×1 k-points for further evaluations of the electronic properties. An equivalent k-point mesh is built for other enlarged cells depending on their sizes.

## 3. Results and Discussion

The various Bi/Al/Ti/Fe/Co/Ni adsorption structures, critical multi-orbital hybridizations, significant NM/AFM/FM, and metallic/semiconducting behaviors are worthy of systematic investigation. The typical adatoms, concentrations, and distributions will clearly illustrate the diverse phenomena.

### 3.1. Bi Adsorptions

**Figure 1:**
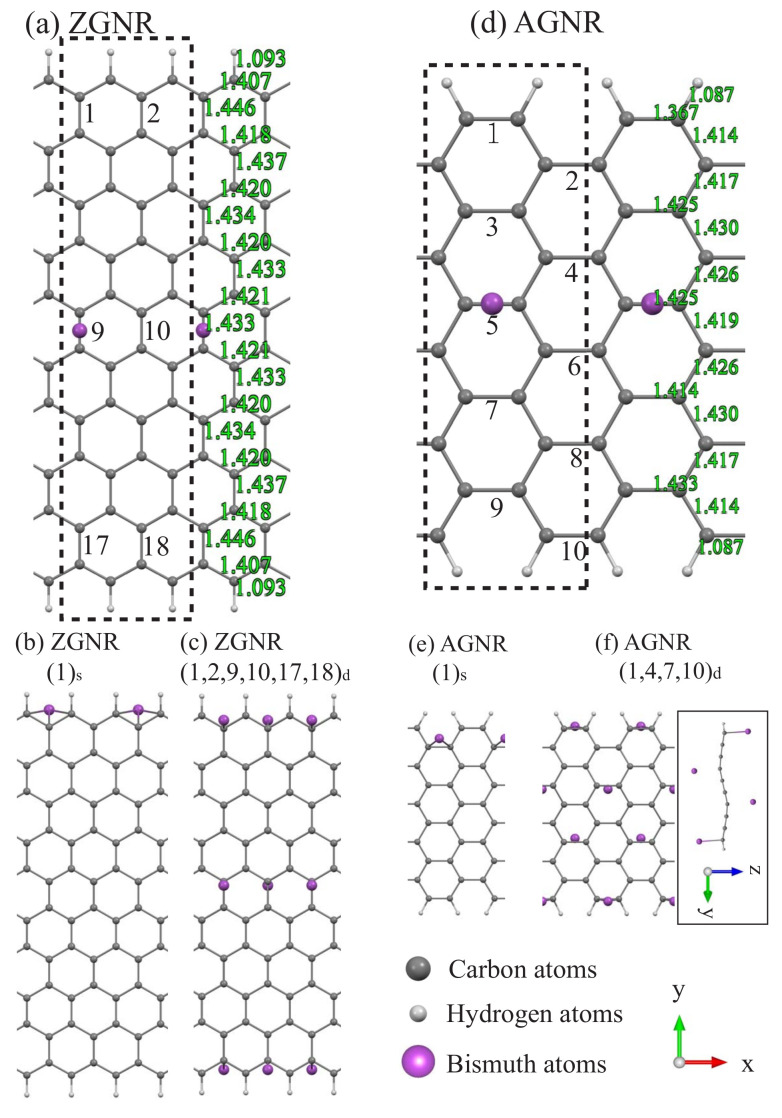
The optimal geometric structures for the Bi-adsorbed $N_{Z}$ = 10 zigzag graphene nanoribbons (ZGNRs) (**a**–**c**) and $N_{A}$ = 10 armchair GNRs (AGNRs) (**d**–**f**), in which the adatom positions are marked by numbers.

Bismuth adatom chemisorption on the GNR surface can create an unusual geometric structure, being sensitive to the distribution and concentration. The optimal adsorption position is located at the bridge site (Figure 1). A single Bi adatom near the ribbon center, e.g., ZGNR (9)${}_{s}$ (Figure 1a) or AGNR (5)${}_{s}$ (Figure 1d), has an adsorption height of about 3.85 (Table 1). When the adatom is close to the ribbon edge, e.g., (1)${}_{s}$, it shifts to the hollow site (along the $\hat{y}$ direction) about 0.3−0.4, and its height is reduced to the range of 2.4−2.5. Similar results are found in the two-atom adsorption cases, e.g., (2,9)${}_{d}$ and (1,17)${}_{s}$ adsorptions. It is very difficult to form the high-concentration adsorption system since the sizes of Bi adatoms are very big, and they have significant Coulomb repulsive interactions within a sufficiently short distance. The maximum concentration is numerically examined to be about four and six Bi adatoms in the $N_{A}=10$ and $N_{z}=10$ systems (Table 1), respectively. The former even leads to the nonplanar/wave-like armchair GNR, indicating the quite strong Bi–C chemical bondings. It is relatively difficult to observe the buckling structure in zigzag systems because of the larger widths. Most of the optimal structures show planar geometries, or a few of them display the wave-like ones. This indicates that the planar σ bondings of carbon atoms are hardly affected by Bi chemisorptions. The above-mentioned significant features clearly indicate the competition/cooperation among Bi–C, C–C, and C–H bonds.

**Figure 2:**
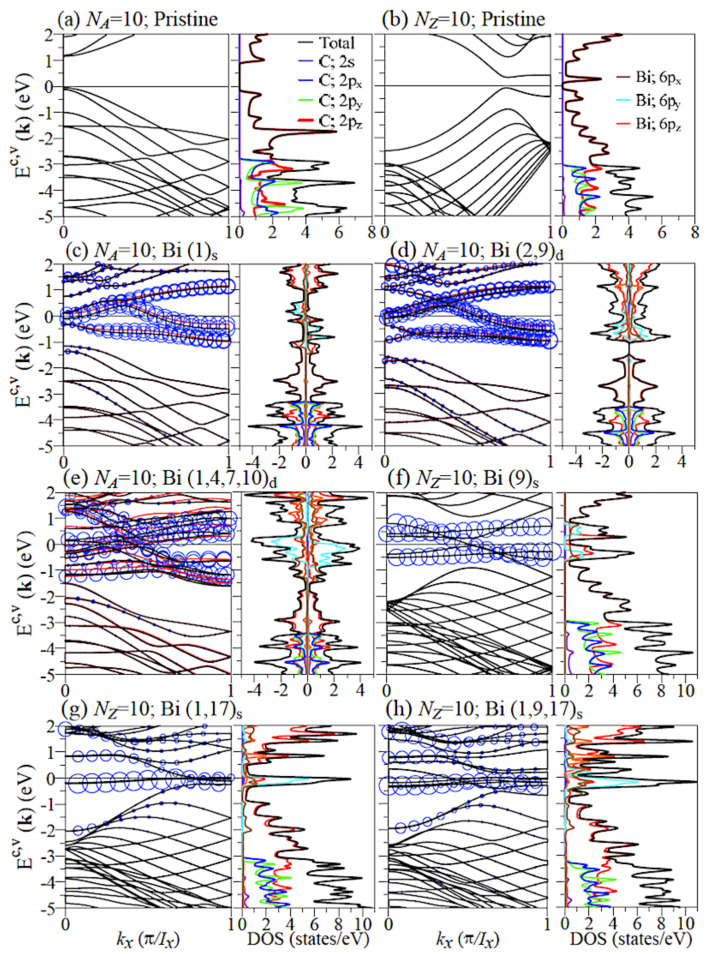
Band structures and densities of state (DOSs) of (**a**) pristine AGNR, (**b**) pristine ZGNR, and Bi-adsorbed GNRs under various coverages: (**c**) (1)${}_{s}$, (**d**) (2,9)${}_{d}$, and (**e**) (1,4,7,10)${}_{d}$ for $N_{A}$=10 armchair systems and (**f**) (9)${}_{s}$, (**g**) (1,17)${}_{s}$, and (**h**) (1,9,17)${}_{s}$ for $N_{Z}$=10 zigzag systems. Superscripts *c* and *v* correspond to the conduction and valence bands, respectively. The blue circles in the band structures represent the contribution of the adatoms.

GNRs exhibit the special 1D band structures owning to the honeycomb lattice symmetry, finite-size quantum confinement, and edge structure. Pristine AGNRs ($N_{A}$ = 10) possess many 1D energy bands, as shown in Figure 2a. The occupied valence bands are asymmetric to the unoccupied conduction bands around the Fermi level ($E_{F}$ = 0), in which a direct energy gap of 1.1 eV arises from the finite-size confinement effect. The low-lying electronic states within ±2 eV and the deeper ones are, respectively, contributed by the π bonds of parallel 2p${}_{z}$ orbitals and the σ bonds of (2s,2p${}_{x}$,2p${}_{y}$) orbitals as indicated by the orbital-projected DOSs. Most of the energy dispersions are parabolic bands, while a few of them are partially flat ones. All the energy dispersions depend on the wave vector monotonously, except for the sub-band anti-crossings. The band-edge states, which occur at k${}_{x}$ = 0, 1, and others related to sub-band anti-crossings, create van Hove singularities in the DOSs. There are certain important differences between zigzag and armchair systems. Pristine ZGNRs ($N_{Z}$ = 10), as shown in Figure 2b, have a pair of partially flat valence and conduction bands nearest to $E_{F}$, corresponding to wave functions localized at the zigzag boundaries. Moreover, these energy bands also exhibit the double degeneracy for the spin degree of freedom. The band-edge states, which appear at k${}_{x}$ = 1/2, determine a direct gap of 0.4 eV as a result of the strong competition between quantum confinement and spin configuration.

**Figure 3:**
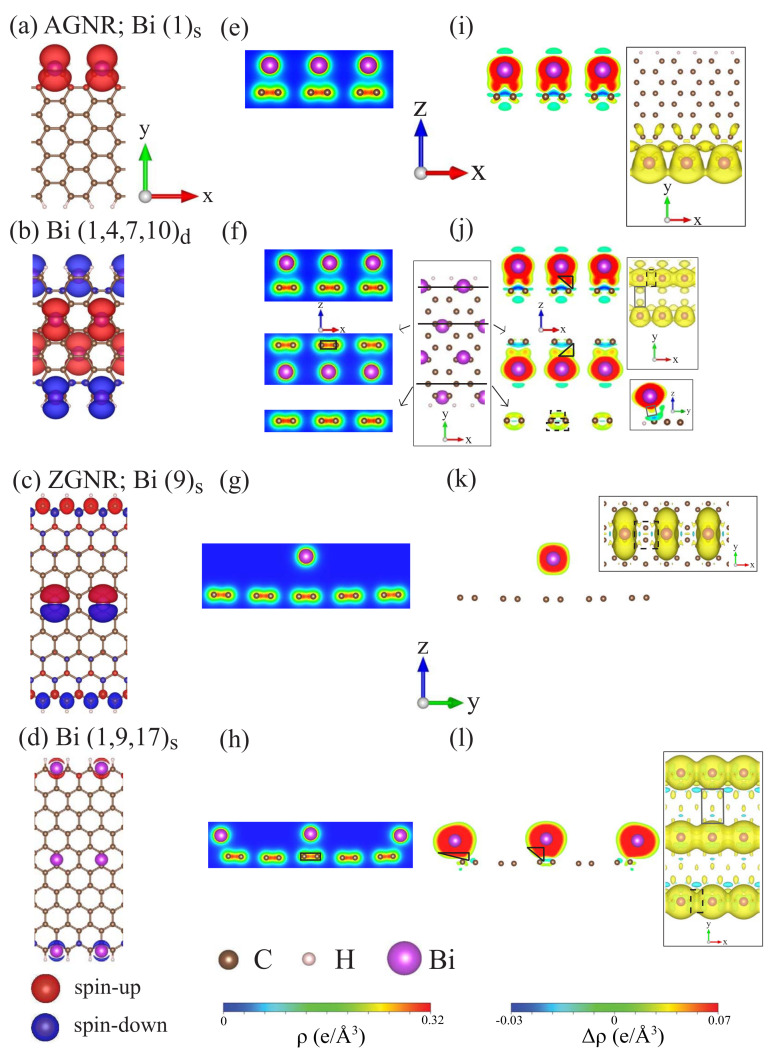
The spin-density distributions for Bi-adsorbed armchair and zigzag GNR systems with (**a**) AGNR(1)${}_{s}$, (**b**) AGNR(1,4,7,10)${}_{d}$, (**c**) ZGNR(9)${}_{s}$, and (**d**) ZGNR(1,9,17)${}_{s}$. Also shown in (**e**–**h**) and (**i**–**l**) are the charge density (ρ) and charge density difference (δρ), respectively.

All the Bi-adsorbed GNR are metals with free carriers arising from the adatom chemisorptions, as clearly revealed in Figure 2c–h. The significant (C, Bi)-co-dominated band structures occur crossing the Fermi level, in which their energy widths increase with the Bi concentration. Moreover, whether band structures exhibit the spin-split behaviors depends on the edge structure and the adatom distribution. All the armchair Bi-adsorbed systems possess the spin-split electronic states, especially for the adatom-dominated energy bands. The spin-up and spin-down distributions near the Fermi level are not equivalent, as clearly indicated by the net magnetic moment in each system (Table 1). The magnetic properties are almost fully determined by the Bi adatoms in the AGNRs. As for the single Bi-adsorbed ZGNRs, there is the absence or existence of magnetism if the Bi adatom is situated at the ribbon center ((9)${}_{s}$) or ribbon edge ((1)${}_{s}$), respectively (Table 1). In general, most of the zigzag systems show non-splitting bands (Figure 2f–h) with a vanishing total magnetic moment. They might belong to AFM or NM configurations, which can be confirmed later by the spin-density distributions (Figure 3a–d). The σ bands ($E^{v}\leq -3$) due to carbon atoms are almost independent of Bi adatoms except for energy shifts, clearly reflecting the optimal geometric structures.

The orbital- and spin-decomposed DOSs obviously reveal the metallic behavior, the magnetic configuration, and the concise orbital hybridizations in chemical bonds. The Bi adatom chemisorptions can induce a finite DOS at the Fermi level (Figure 2c–h), in which its value directly reflects the energy dispersions crossing $E_{F}=0$. Most cases exhibit very high DOSs (Figure 2d–h), accompanied by the weakly dispersive bands. The spin-up and spin-down DOSs are different in all the Bi-adsorbed GNRs except for the symmetric adatom distributions in zigzag systems (Figure 2f–h). Their difference in the covered area means the FM configuration is proportional to the net magnetic moment. As for the orbital hybridizations, the DOSs arising from the (2s,2p${}_{x}$,2p${}_{y}$) orbitals of carbon atoms (E≤−3 eV) are hardly affected by the surface adsorptions. This means the planar σ bonding almost keeps similar, being consistent with the optimal geometric structures. Only the 2p${}_{z}$ orbitals might take part in the Bi-C bonding, observed by the van Hove singularities between C(2p${}_{z}$) and Bi (6p${}_{x}$,6p${}_{y}$,6p${}_{z}$) orbitals. The 6p${}_{z}$-2p${}_{z}$ bondings are strongest among three kinds of orbital hybridizations in Bi–C bonds, as clearly indicated from the strength and number of combined van Hove singularities. However, the emergence is greatly reduced in the single-adatom central adsorptions, e.g., (9)${}_{s}$ in zigzag systems (Figure 2f), as a result of the very weak Bi–C bonding. As for the the significant hybridizations of (6p${}_{x}$,6p${}_{y}$,6p${}_{z}$) in Bi–Bi bondings, they are obviously revealed in the three-orbital-dependent merged structures, in which the intensities are enhanced in the increase of adatom concentration.

The spin configuration (Figure 3a–d) and the strength of the magnetic moment (Table 1) are greatly diversified by the Bi chemisorptions on the GNR surfaces. The isolated Bi atoms could themselves induce the intrinsic magnetic moment, so only the FM and AFM spin distributions come to exist after Bi adsorptions. All the armchair systems show the FM configurations, while the zigzag ones exhibit the FM/AFM spin arrangement. For the former, the Bi guest atoms create the spin-up distribution, also leading to the minor and similar magnetic configuration of the neighboring C atoms (Figure 3a,b). The magnetism is strongly related to the chemical environment experienced by the Bi adatoms. Especially for zigzag systems, when the Bi-adsorbed system retains the symmetric geometry under the single adsorptions, the adatom simultaneously exhibits the spin-up and spin-down magnetic moments with the same magnitude ((Figure 3c), or the Bi-dependent magnetism thoroughly vanishes ((Figure 3d). However, the Bi adatom in an asymmetric edge distribution itself only generates the spin-up state, e.g., (1)${}_{s}$ in Table 1. The magnetic moments are also very sensitive to the adatom concentration and the terminated C–H bonds at the edge positions.

The charge density distribution and its variation can provide very useful information about the orbital bondings and charge transfer. In general, the Bi adatoms possess the (6p${}_{x}$,6p${}_{y}$,6p${}_{z}$) orbitals, which will take part in the Bi–C and Bi–Bi bonds. The multi-orbital hybridizations are clearly revealed in the spatial charge distributions, as observed in Figure 3e–l. The single adatom at central adsorption is shown in Figure 3g,k, in which ρ and Δρ, respectively, almost have no charge distributions between guest and host atoms. This suggests the rather weak interactions in Bi–C bonds due to the large adsorption height (Table 1). On the other hand, for most of the Bi adatom adsorptions, there exist the observable charge distributions in Bi–C bonds and more complicated orbital interactions in Bi–Bi bonds illustrated in the xz- and xy-planes. That is, the asymmetric single-adatom and higher concentration adsorptions could create more orbital hybridizations in Bi–C and Bi–Bi bonds (for the high Bi concentration systems), such as (1)${}_{s}$ and (1,4,7,10)${}_{d}$ in armchair systems and (1,9,17)${}_{d}$ in zigzag cases. The significant Bi–C bondings induced the charge distributions between them, accompanied by the partial distortions of the π bondings. The distorted and extended π bondings and the Bi–Bi bonds dominate the metallic behavior, especially for the latter. This is consistent with the orbital-projected DOSs discussed in Figure 2. The important orbital hybridizations in Bi–C bonds, as observed from the charge differences, cover 6p${}_{z}$-2p${}_{z}$, 6p${}_{z}$-2p${}_{y}$, and 6p${}_{z}$-2p${}_{x}$, in which the first kind has the strongest orbital hybridization among them.

### 3.2. Al Adsorptions

**Figure A1:**
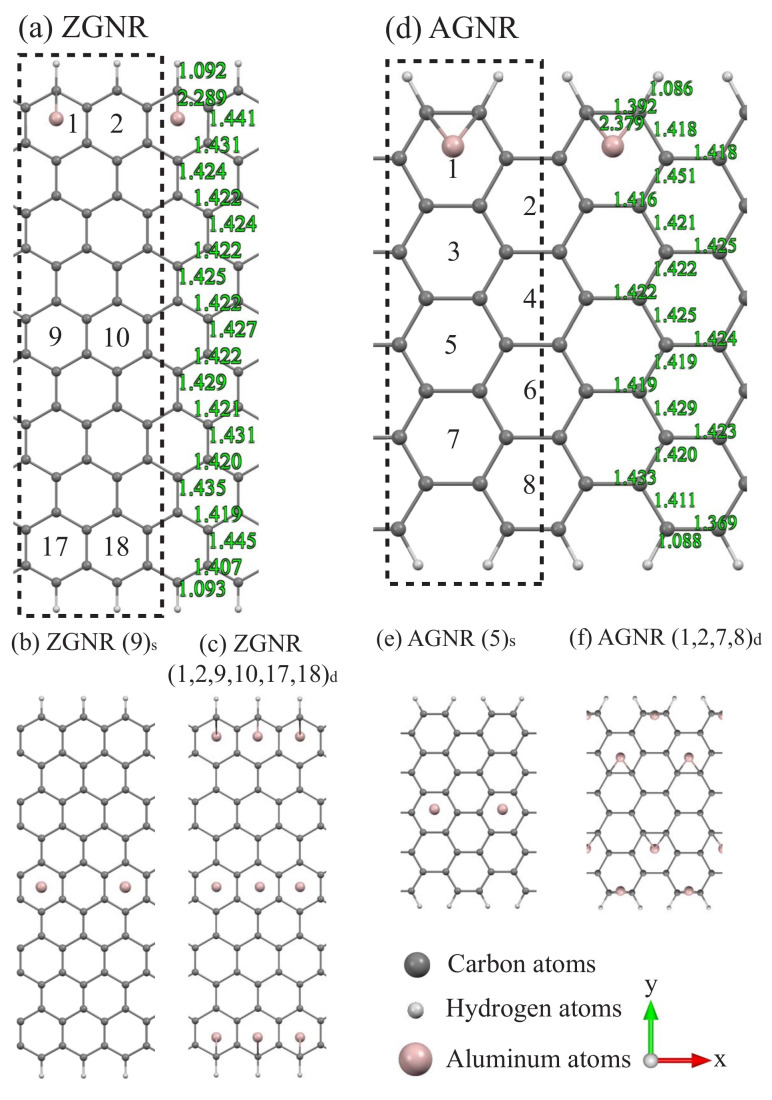
The optimal geometric structures for the Al-adsorbed $N_{Z}$ = 10 ZGNRs (**a**–**c**) and N${}_{A}$ = 10 AGNRs (**d**–**f**), in which the adatom positions are marked in numbers.

The Al-adsorbed GNR remains the planar structure with a non-uniform honeycomb lattice, as revealed in Figure A1. This clearly indicates that the well-behaved σ bondings of carbon atoms are hardly affected by the Al adsorptions. The optimal positions might correspond to the hollow sites if the adatoms are far away from the boundaries, i.e., the xy-plane projections are the centers of the hexagon lattice in the absence of y-shift (Table 1). However, when the Al adatoms are located near the armchair and zigzag boundaries, the obvious shifts are revealed along the transverse direction toward the edges. The height of the Al adatom is 2.07–2.2/2.00–2.14 for the armchair/zigzag systems, respectively, in which the maximum value is associated with the adsorption at the center of the ribbon. That is, the Al adatoms are relatively low under the effect of the edge C–H bonds. As for the highest concentration, the stable structure is associated with the double-side adsorption of four Al adatoms in the $N_{A}=10$ armchair system, and it is related to the similar adsorption of six Al adatoms in the $N_{z}=10$ zigzag one. This result does not exceed the upper limit of 25% in Al-adsorbed monolayer graphene [36]. There exist unusual geometric structures under the maximum concentration, as obviously indicated in Figure A1. The optimal position is dramatically transferred to the bridge site, clearly illustrating the complex competitions/cooperations among the C–C, Al–C, Al–Al, and H–C chemical bondings. This means that the two Al adatoms need to have a sufficiently long distance to achieve a stable structure. After Al adsorption, the similar 1D graphene plane suggests that only the 2p${}_{z}$ orbitals of carbons have significant chemical bondings with the adatoms, and the σ bondings of (2s,2p${}_{x}$,2p${}_{y}$) almost keep the same. This will be explored thoroughly.

**Figure 4:**
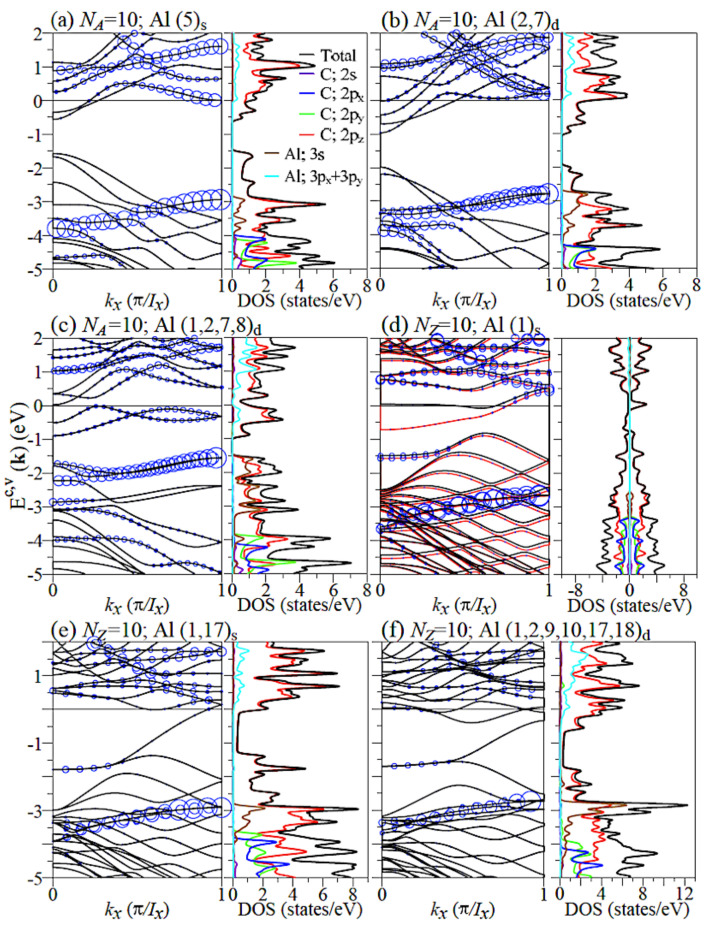
Band structures and DOSs of Al-adsorbed GNRs under various coverages: (**a**) (5)${}_{s}$, (**b**) (2,7)${}_{d}$, and (**c**) (1,2,7,8)${}_{d}$ for $N_{A}$=10 armchair systems and (**d**) (1)${}_{s}$, (**e**) (1,17)${}_{s}$, and (**f**) (1,2,9,10,17,18)${}_{d}$ for $N_{Z}$=10 zigzag systems. The blue circles in band structures represent the contribution of the adatoms.

**Figure A2:**
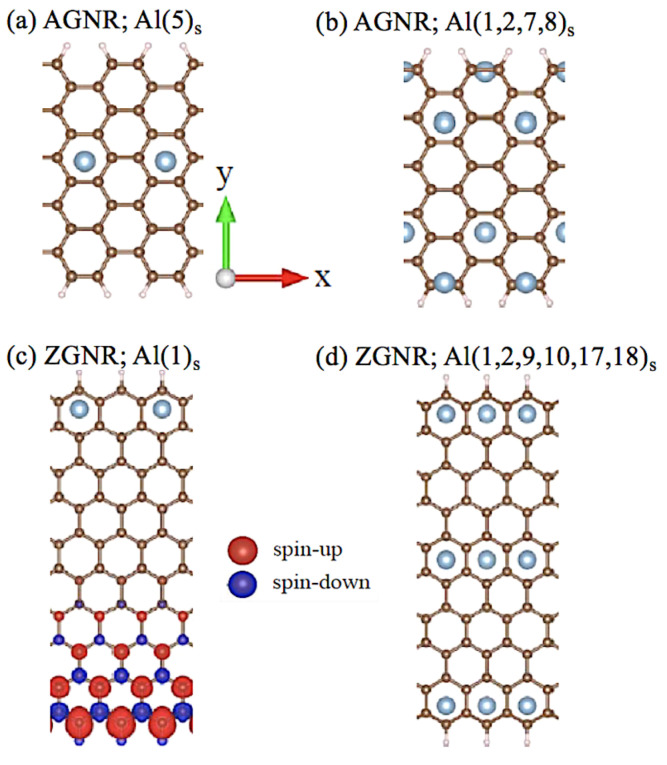
The spin-density distributions for Al-adsorbed armchair and zigzag GNR systems with (**a**) AGNR(5)${}_{s}$, (**b**) AGNR(1,2,7,8)${}_{d}$, (**c**) ZGNR(1)${}_{s}$, and (**d**) ZGNR(1,2,9,10,17,18)${}_{s}$.

The electronic structures exhibit the drastic changes in all GNRs, sensitive to the changes in edge structures, Al-adsorbed position, and concentration, but not the single- or double-side adsorption (Figure 4a–f). In general, the Fermi level is shifted from the center of the energy gap to the conduction bands. Furthermore, the energy bands very close to $E_{F}$ are mainly determined by carbon atoms, indicating the free electrons due to the distorted π bondings and the charge transfer from carbon atoms to Al adatoms. With higher Al concentrations, there are more carbon-dominated conduction bands intersecting with the Fermi level, leading to the increase in the 1D linear carrier density. In most cases, the Al adatoms make important contributions to certain conduction and valence bands in the range of $0\leq \;E^{c}\leq \;2.0$ eV and −2.5 eV $\geq \;E^{v}\geq \;-4.3$ eV, in which the −2.5 eV $\geq \;E^{v}\geq \;-3.5$ eV valence states are weakly dispersive and almost doubly degenerate. The Al adsorption could create the extra band-edge states arising from the subband hybridizations. Specifically, for zigzag systems, whether the low-lying energy bands exhibit the spin spitting is very sensitive to the adsorption position of Al, which can be verified by the spin-density distributions (Figure A2).

The Al adatom adsorptions can create the metallic DOSs except the maximum concentration (Figure 4), in which the main characteristics of van Hove singularities are in sharp contrast with those of pristine systems (Figure 2a,b). The low-energy DOSs are dominated by the 2p${}_{z}$ orbitals of carbons, being consistent with the atom-dominated band structures. This feature clearly illustrates that the π bondings are distorted only near the Al adatom sites, and they behave like the normal extended states at other positions. There exist the specific zero DOSs below the Fermi level about the range of −2 eV $\leq \;E^{v}\leq \;-1$ eV, belonging to the initial valence and conduction bands. Concerning the clear evidence of the multi-orbital hybridizations in Al–C bonds, they show as the merged peaks in the range of E<−1.5 eV from the Al-3s orbital and the C-2p${}_{z}$ orbital and those for E>−0.5 eV due to the Al-(3p${}_{x}$,3p${}_{y}$) orbitals and the C-2$p_{z}$ orbital. Energy bandwidths of the 3s and (3p${}_{x}$ + 3p${}_{y}$) orbitals grow gradually in the increment of Al concentrations, indicating two kinds of orbital hybridizations in Al–Al bonds. The 3p${}_{z}$ orbitals of Al adatoms hardly contribute to the significant chemical adsorption, since each adatom only has three occupied orbitals in the outermost ones, in which it might possess two states in the 3s orbitals and one state in the (3p${}_{x}$,3p${}_{y}$) orbitals.

**Figure 5:**
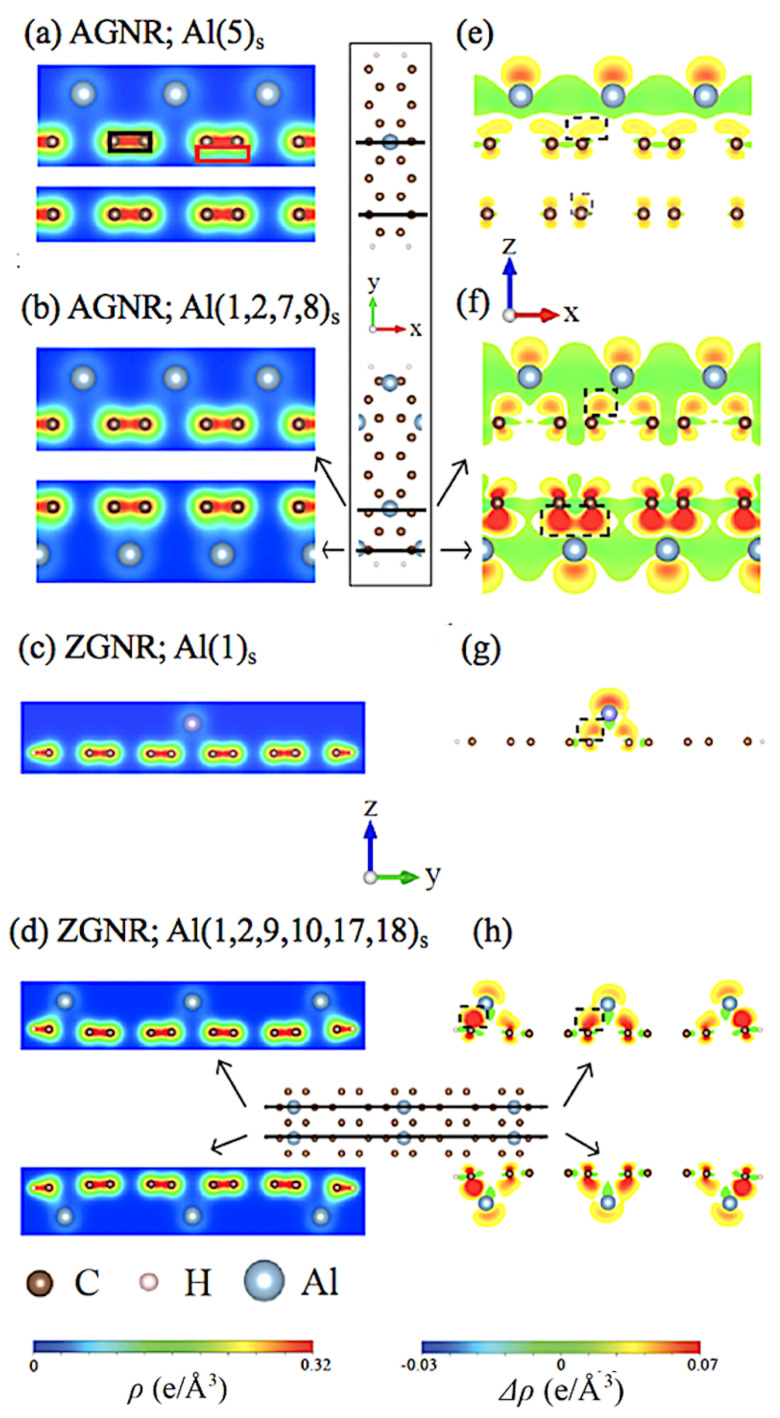
The charge density (ρ) for Al-adsorbed armchair and zigzag GNR systems with (**a**) AGNR(5)${}_{s}$, (**b**) AGNR(1,2,7,8)${}_{d}$, (**c**) ZGNR(1)${}_{s}$, and (**d**) ZGNR(1,2,9,10,17,18)${}_{s}$. Shown also in (**e**–**h**), the corresponding charge density difference (δρ).

The group-V elements have three valence electrons in the outermost orbitals, and they can form the complex multi-orbital hybridizations after chemisorptions on GNR surfaces. The bonding strength of C–C, Al–C, and Al–Al bonds are revealed in Figure 5a–h. After the Al adatom adsorptions, all the C–C bonds possess the strong covalent σ bonds (black rectangle) and the somewhat weakened π bonds simultaneously (red rectangle). The former almost keep the same; furthermore, the π bonding also belongs to the extended state in a 1D system except that it is seriously distorted under the maximum concentration case (Figure 5f). These are responsible for the carbon-dominated low-lying energy bands with metallic or semiconducting behavior. In general, the 3s-orbital electrons (black triangle) are redistributed between Al and the six nearest C atoms, revealing a significant hybridization with the 2p${}_{z}$ orbitals. The electrons of the Al adatom move to the top and bottom of the non-nearest C atoms, inducing free electrons in conduction bands. The higher the Al concentration is, the more electrons are transferred.

### 3.3. Ti/Fe/Co/Ni Adsorptions

**Figure A3:**
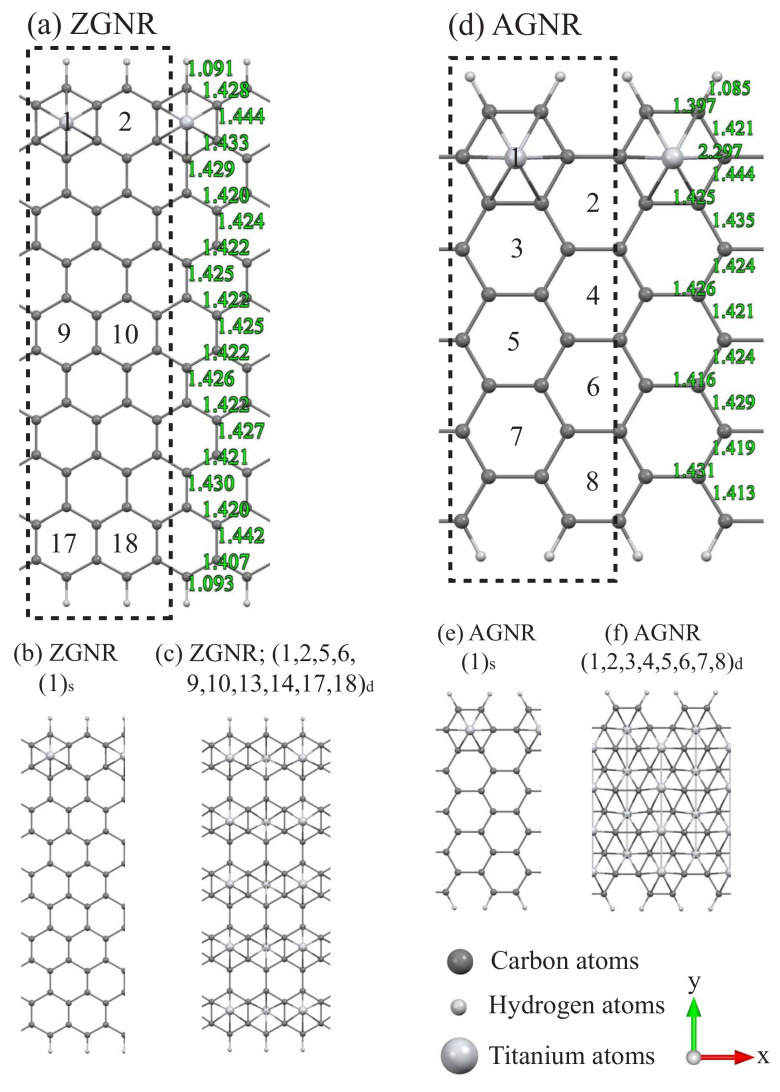
The optimal geometric structures for the Ti-adsorbed $N_{Z}$ = 10 ZGNRs (**a**–**c**) and $N_{A}$ = 10 AGNRs (**d**–**f**), in which the adatom positions are marked in numbers.

Similar to the Al adatom, transition metals (Ti, Fe, Co, Ni) are also preferred to be adsorbed at the hollow-site (Figure A3), which is consistent with previous studies [23,37,38]. However, there are no shifts relative to the (x,y)-projection centers under all the adsorption configurations. This is in sharp contrast with the significant shifts of the Al and Bi metal adsorptions. The Ti heights keep in the short range of ∼1.50–2.10 Å, leading to the largest binding energies among the (Ti, Al, Bi) adatoms. This means it is relatively easy to induce the Ti chemisorptions, compared with the Al and Bi adatoms. The main features of the optimal structures strongly suggest that the 2p${}_{z}$ and (2s,2p${}_{x}$,2p${}_{y}$) orbitals of carbon atoms, respectively, make important and minor contributions to the Ti–C bonds. For Fe/Co/Ni systems, the adatom heights are in the range of ∼1.40–2.00 Å. Their binding energies are larger under higher concentrations (Table 1). It is relatively easy to induce the high concentration of Fe chemisorptions, compared with the above-mentioned metal adatoms. The Fe adatoms could reach higher adatom concentrations, as well as Ti adatoms. Specifically, the buckling structures might be revealed for the highest-concentration adsorption in armchair/zigzag systems, which are similar to Ti adsorptions.

**Figure 6:**
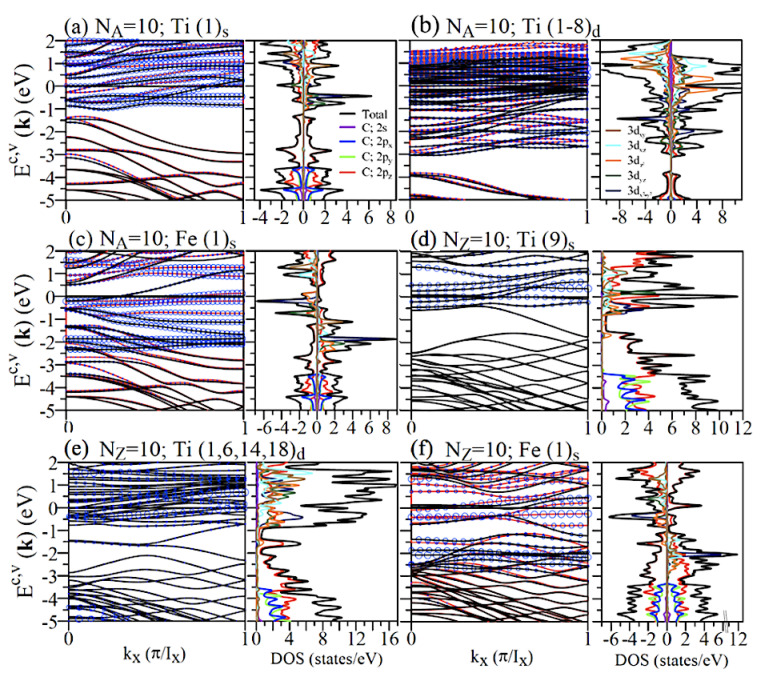
Band structures and DOSs of Ti-/Fe-/Co-/Ni-adsorbed GNRs under various coverages: (**a**) Ti(1)${}_{s}$, (**b**) Ti(1-8)${}_{d}$, and (**c**) Fe(1)${}_{s}$ for $N_{A}$ = 10 armchair systems and (**d**) Ti(9)${}_{s}$, (**e**) Ti(1,6,14,18)${}_{d}$, and (**f**) Fe(1)${}_{s}$ for $N_{Z}$ = 10 zigzag systems. The blue circles in band structures represent the contribution of the adatoms.

**Figure A4:**
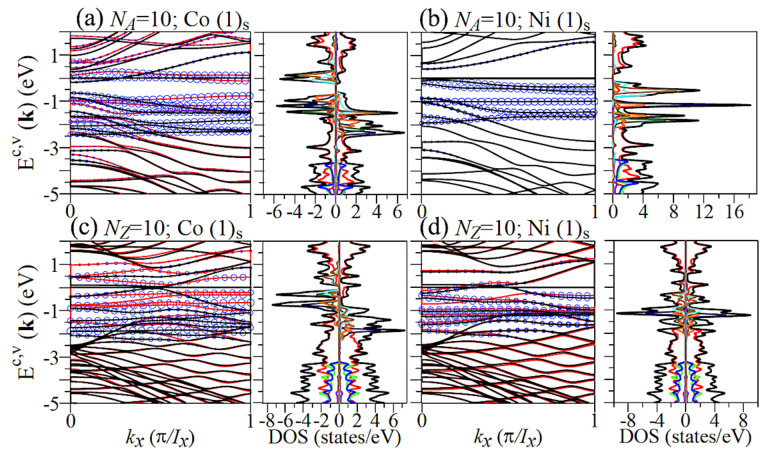
Band structures and DOSs of Co/Ni-adsorbed GNRs: (**a**) Co(1)${}_{s}$ and (**b**) Ni(1)${}_{s}$ for $N_{A}$ = 10 armchair systems and (**c**) Co(1)${}_{s}$ and (**d**) Ni(1)${}_{s}$ for $N_{Z}$ = 10 zigzag systems. The blue circles in band structures represent the contribution of the adatoms.

All the Ti-/Fe-/Co-/Ni-adsorbed GNRs belong to the unusual metals, as clearly indicated in Figure 6 and Figure A4. There are many energy bands crossing the Fermi level, in which they mainly come from both adatoms and carbon atoms. Furthermore, the adatom-dominated energy bands might be thoroughly occupied or unoccupied. It is worth mentioning that the Ti/Fe/Co/Ni chemisorptions could induce the diversified energy dispersions because of the edge structures and adatom types. For armchair systems, all of them exhibit the spin-split energy bands, especially for those near $E_{F}$. Apparently, they correspond to the FM spin configurations, and the net magnetic moments due to the Ti/Fe adsorbates are sensitive to the adatom distribution and concentration (Table 1). The Ti-/Fe-dominated electronic structures lie in the range of −1 eV $\leq \;E^{c,v}$ / −3 eV $\leq \;E^{c,v}$, and the energy bands below it are fully determined by the host C atoms. The Ti/Fe adatoms hardly contribute to the deep valence states, indicating the weak chemical hybridizations from the (2s,2p${}_{x}$,2p${}_{y}$) orbitals in the adatom-C bonds (Figure 6a–c). For zigzag systems, the partially flat bands across the Fermi level only survive under the single-adatom central adsorption (Figure 6). However, most of the chemisorption cases lead to dramatic changes in the low-lying energy bands because of the significant adatom-edge-C bondings; that is, the edge-C-dominated partially flat energy dispersions are thoroughly absent. The zigzag systems might be FM, AFM, and NM metals, in which the latter two need to be further examined from the spin-density distributions.

The orbital- and spin-projected DOSs in Ti-/Fe-/Co-/Ni-adsorbed GNRs could provide the very complicated van Hove singularities and thus identify the significant multi-orbital hybridizations in Ti–C and Ti–Ti bonds. For any chemisorptions, there is an obvious DOS at the Fermi level, directly indicating the creation of the high free carrier density. This is closely related to carbon guest atoms and the Ti guest adatom. The spin-split DOSs are revealed in most adsorption cases except for the symmetric adatom distributions in zigzag systems (Figure 6d,e). The results further illustrate the adatom-induced spin states and their strong competitions with edge-carbon magnetic arrangement by the adatom-C chemical bondings. As for the various orbital contributions to DOSs, the 2s, 2p${}_{x}$, and 2p${}_{y}$ of carbons appear at E≤−3.4 eV, implying that their interactions with the outer five 3d orbitals of Ti adatoms should be weak. However, the 2p${}_{z}$ orbitals experience rather high hybridizations with the 3d${}_{xy}$, 3d${}_{xz}$, 3d${}_{yz}$, 3d${}_{z^{2}}$ and 3d${}_{x^{2}-y^{2}}$, since the special structures of DOSs are seriously merged together in the range of −1 eV ≥E≥3 eV/−2 eV ≤E≤3 eV for the low/high adsorptions. The Ti–Ti bonding is displayed in the enhancement of the metallic 3d-bandwidth.

**Figure 7:**
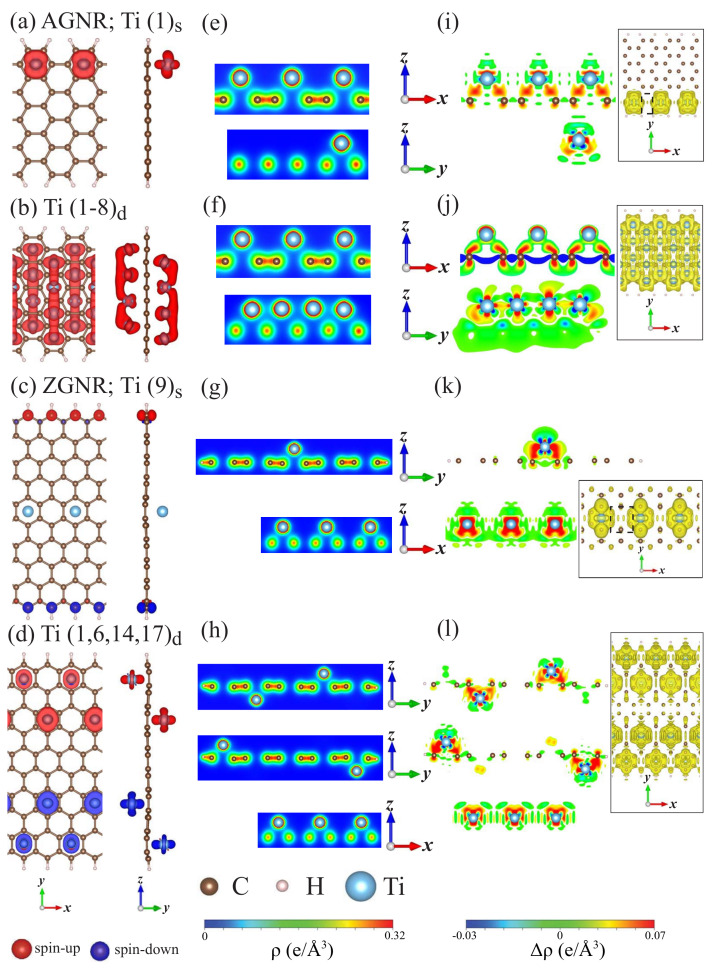
The spin-density distributions for Ti-adsorbed armchair and zigzag GNR systems with (**a**) AGNR(1)${}_{s}$, (**b**) AGNR(1-8)${}_{d}$, (**c**) ZGNR(9)${}_{s}$, and (**d**) ZGNR(1,6,14,18)${}_{d}$. Shown also in (**e**–**h**) and (**i**–**l**) are the charge density (ρ) and charge density difference (δρ), respectively.

There exist diverse FM and AFM spin distributions. Any armchair systems have the distinct FM configurations, being sensitive to the single- and double-side adsorptions and concentrations. The single-adatom chemisorptions, e.g., Ti(1)${}_{s}$ (Figure 7a), could create the comparable magnetic moments (∼1.52 μB in Table 1). In general, the strength of the magnetic response is proportional to the Ti concentration under the specific double-side adsorptions. The FM and AFM configurations, respectively, correspond to the asymmetric ((1)${}_{s}$ in Figure 7a) and symmetric ((9)${}_{s}$ in Figure 7c) adatom distributions in zigzag systems. The guest adatom, which is located at/near the zigzag boundary, can even fully destroy the spin-dominated arrangement from the neighboring carbons (Figure 7d). This is purely due to the strong competition of edge carbon atoms and adatoms, similar to the Bi and Al cases.

The chemical bonding between Ti and C is obvious and significant even for the single-adatom cases, regardless of the positions, such as (1)${}_{s}$ and (9)${}_{s}$ for $N_{A}=10$ and $N_{z}=10$, respectively (Figure 7e,g). Its strength grows with the increasing adatom concentration, e.g., ${\left(1-8\right)}_{d}$ and ${\left(1,6,14,17\right)}_{d}$ (Figure 7f,h). For any adsorption cases, large charge transfers exist between Ti guest adatoms and carbon host atoms on the xz- and yz-planes, as clearly revealed in Figure 6i–l. It can only identify the significant orbital hybridizations of (3d${}_{xz}$,3d${}_{yz}$,3d${}_{xy}$,3d${}_{z^{2}}$,3d${}_{x^{2}-y^{2}}$) five orbitals and the 2p${}_{z}$ orbital. As for the Ti–Ti chemical bondings, the charge distributions are easily observed only on the yz-plane at a high concentration of armchair systems, e.g., ${\left(1-8\right)}_{d}$. The charge differences become obvious for any chemisorptions on the xz-plane and/or the yz-plane; furthermore, there are charge extensions and even overlaps on the xy-plane at the optimal heights of Ti adatoms. The observable five orbital hybridizations in Ti–Ti bonds are responsible for the low-lying Ti-dominated energy bands, being also one of the critical factors in the creation of the conduction electron density/the metallic behavior.

### 3.4. Proposed Experimental Verifications and Potential Applications

The main features of band structures and DOSs could be examined by angle-resolved photoemission spectroscopy (ARPES) and scanning tunneling spectroscopy (STS) measurements. ARPES is an effective tool in identifying the diverse band structures of graphene-based systems, e.g., the gapless valence Dirac cone in monolayer graphene [39] and the 1D parabolic valence bands near the Γ point accompanied by band gaps and distinct energy spacings of AGNRs in the presence/absence of hydrogen passivation [40,41]. Furthermore, ARPES has been used to verify the effects arising from doping. The greatly modified band structures of the Ti-absorbed graphene [42] or the red shift of 1–1.5 eV in the pi bands (*n*-type doping) [43] has been confirmed by using high-resolution ARPES. On the other hand, STS measurements of the d*I*/d*V* spectra have served to confirm the width- and edge-dominated energy gaps and the asymmetric peaks of 1D parabolic bands of GNRs [44,45,46]. Moreover, the Fermi level red shift of Bi-doped graphene has also been observed by STS [47]. Further, ARPES and STS examinations are desirable for the aforementioned main structures of the electronic properties of metal/transition metal-adsorbed GNR systems. In addition, it could pave the way for tight-binding model research in determining the important hopping integrals. According to the detailed analyses on the low-lying band-edge states and energy dispersions along with the high-symmetry points, these two methods could present almost the same band structures. Apart from this, high-resolution STM [48] and transmission electron microscopy (TEM) [49] could be utilized to verify the geometric structure predictions.

Recently, nanospintronics has attracted much research attention due to its remarkable advantages for next-generation information processing and data storage technologies [50,51,52,53]. Finding the way to effectively induce magnetic ordering or magnetic moments plays an important role in nanospintronic applications. In our work, the spin arrangement and magnetic moments are strongly associated with the edge carbons and adsorbates. Al adatoms do not create the spin distributions, but could affect the GNR zigzag edges, leading to the AFM/FM/NM configurations for the adsorptions at the center, one edge, or both edges of the carbon ribbon, respectively. On the other hand, most of Bi- and Ti-/Fe-/Co-adsorbed asymmetric systems exhibit the FM configurations under the induced spin states themselves and the greatly reduced edge-carbon magnetic moments, being in sharp contrast with the pristine GNR magnetic configuration. Specifically, the strong magnetic moments of Ti-, as well as Fe-adsorbed systems (Table 1) might bring potential applications in spintronic devices [50,54,55]. There are several efficient methods for detecting magnetic moments [56]. The first one is using a superconducting quantum interference device, which successful detects the paramagnetism in defect fluorinated graphene. The second method is via spin transport measurements that were firstly used to detect the magnetic moment formation in hydrogenated graphene. Moreover, magnetic moments in GNR could also be measured by magnetic force microscopy or spin-polarized STM.

## 4. Conclusions

In general, the metal (Bi, Al)- and transition metal (Ti, Fe, Co, Ni)-adsorbed GNRs can greatly induce metallic behaviors. They clearly display important differences in their fundamental properties. The Al and Ti/Fe/Co/Ni guest atoms exhibit the hollow-site optimal positions, while the former might have the y-direction shifts, especially for the non-symmetric distributions. The similar shifts are revealed in the deviated bridge-site Bi adsorptions. The adatom chemisorptions are relatively easily observed in the Ti systems with the largest binding energies. Energy bands, which cross the Fermi level, mainly arise from carbon atoms or metal atoms for Al-adsorbed systems or the Bi/Ti/Fe/Co/Ni ones, respectively. Three types of observable Bi–C bondings, 6p${}_{z}$-2p${}_{z}$, 6p${}_{x}$-2p${}_{z}$, and 6p${}_{y}$-2p${}_{z}$, are characterized by the charge distributions and DOSs except for the symmetric single-adatom adsorptions. The important (6p${}_{x}$, 6p${}_{y}$, 6p${}_{z}$)-(6p${}_{x}$, 6p${}_{y}$, 6p${}_{z}$) orbital hybridizations in Bi-Bi bonds are responsible for the Bi-induced low-lying energy bands. For Al adsorptions, there exist the adatom-dominated valence bands at $E_{v}∼$3 eV and the partial adatom contributions to the conduction bands, being consistent with the 3p${}_{z}$-2p${}_{z}$ and (3p${}_{x}$+3p${}_{y}$)-2p${}_{z}$ orbital hybridizations in Al–C bonds. This feature is also supported by the spatial charge distributions and DOSs. The multi-orbital hybridizations between Ti/Fe/Co/Ni-C bonds are very complicated, in which five orbitals of the adatom, (d${}_{xy}$, d${}_{xz}$, d${}_{yz}$, d${}_{z^{2}}$, d${}_{x^{2}-y^{2}}$), have strong interactions with 2p${}_{z}$ orbitals of carbons. Such orbitals also take part in the adatom-adatom chemical bondings.

As for the magnetic properties, the spin arrangement is strongly associated with the edge carbons and adsorbates. The Al adatoms do not create the spin distributions, but their interactions with the zigzag carbon atoms can destroy the latter’s and thus create the FM or NM configurations. Armchair and zigzag Al-adsorbed GNRs, respectively, belong to the NM and AFM/FM/NM metals. On the other side, most of the Bi- and Ti/Fe/Co-adsorbed asymmetric systems exhibit the FM configurations under the induced spin states themselves and the greatly reduced edge-carbon magnetic moments. Furthermore, the symmetric adatom distributions in zigzag systems might lead to the very complicated AFM configurations, being in sharp contrast with the pristine magnetic configuration. The predicted geometric structures, electronic properties, and magnetic configurations are worthy of further experimental examinations. This work should serve as a first step towards a further investigation into other essential properties of emergent materials for potential applications. Moreover, the theoretical framework proposed in this paper could be generalized to other chemisorption 1D and 2D graphene-related materials.

## Figures and Tables

**Table 1:** The calculated adatom height, adatom y-shift, binding energies, magnetic moment/magnetism for (metals. transition metals)-adsorbed $N_{A}$ = 10 armchair and $N_{Z}$ = 10 zigzag GNRs under various distributions and concentrations. NM, nonmagnetic.

	Configurations	Height (Å)	Adatom y-Shift (Å)	$E_{b}$ (eV)	*M* ($\mu_{B}$)
AGNR	Pristine	-	-	-	0/NM
	Bi(1)${}_{s}$	2.397	0.502	−0.786	0.56/FM
	Bi(5)${}_{s}$	3.854	-	−0.033	1.18/FM
	Bi(2,9)${}_{d}$	2.442	0.571	−0.024	0.21/FM
	Bi(1,2,9,10)${}_{d}$	3.319	1.011	−0.615	1.35/FM
	Bi(1,4,7,10)${}_{d}$	3.046	0.154	−0.276	0.21/FM
	Al(1)${}_{s}$	2.071	0.319	−0.641	0/NM
	Al(5)${}_{s}$	2.207	-	−0.639	0/NM
	Al(2,7)${}_{s}$	2.077	-	−0.453	0/NM
	Al(2,7)${}_{d}$	2.065	-	−1.969	0/NM
	Al(1,2,7,8)${}_{d}$	2.153	0.144	−0.714	0/NM
	Ti(1)${}_{s}$	1.777	-	−2.828	1.28/FM
	Ti(5)${}_{s}$	1.784	-	−2.828591	1.52/FM
	Ti(1-8)${}_{d}$	1.822	-	−3.454	5.93/FM
	Fe(1)${}_{s}$	1.606	0.018	−1.125	2.37/FM
	Fe(2,7)${}_{d}$	1.596	0.003	−2.529	4.78/FM
	Co(1)${}_{s}$	1.566	0.026	−1.291	1.39/FM
	Ni(1)${}_{s}$	1.602	0.013	−1.311	0/NM
ZGNR	Pristine	-	-	-	0/AFM
	Bi(1)${}_{s}$	2.325	1.648	−1.142	0.54/FM
	Bi(9)${}_{s}$	3.889	-	−0.319	0/AFM
	Bi(1,17)${}_{s}$	2.26	1.653	−1.233	0/AFM
	Bi(1,9,17)${}_{s}$	2.294	1.536	−0.909	0/AFM
	Al(1)${}_{s}$	2.049	0.172	−1.606	0.56/FM
	Al(5)${}_{s}$	2.076	0.064	−1.074	0.18/FM
	Al(9)${}_{s}$	2.135	-	−1.142	0/AFM
	Al(1,17)${}_{s}$	1.999	0.317	−1.789	0/NM
	Al(1,2,9,10,17,18)${}_{d}$	2.142	0.167	−0.706	0/NM
	Ti(1)${}_{s}$	1.711	-	−2.774	1.46/FM
	Ti(9)${}_{s}$	1.629	-	−1.991	0/AFM
	Ti(1,6,14,17)${}_{d}$	1.607	-	−2.354	0/AFM
	Ti(1,2,5,6,9,10,13,14,17,18)${}_{d}$	1.665	-	−2.606	0/AFM
	Fe(1)${}_{s}$	1.659	0.005	−1.227	2.4/FM
	Co(1)${}_{s}$	1.568	0.011	−1.844	1.07/FM
	Ni(1)${}_{s}$	1.621	0.015	−1.879	0.019/FM

## Data Availability

The data presented in this study are available in this article.

## Appendix A

It is worth mentioning that there are various functionals to calculate the exchange-correlation energy terms. The original functional is local density approximation (LDA), which depends just on the value of the density at a point so that the results are not accurate enough. A correction to LDA can be obtained by generalized gradient approximation (GGA) functionals that consider the electron density, as well as its gradients. These not only depend on the value of the density at a point (as in the case of LDA), but also on its gradient. In addition, another approach is using hybrid functionals, the mixtures of DFT and Hartree–Fock exchange energies. This hybrid method has similar or a little higher accuracy compared to GGA, but is very time consuming. In our work, we chose GGA, in particular PBEGG,A due to its accuracy and less time consumption.

The binding energy (Table 1), which characterizes the lowered total ground state energy after the chemical adsorption, is expressed as E${}_{b}$ = (E${}_{sys}$− E${}_{GNR}$− nE${}_{A}$)/n, where E${}_{sys}$, E${}_{GNR}$, and E${}_{A}$ are the total energies of the adsorbed system, the GNR sheets, and the isolated adatoms, respectively.

In great contrast with the FM behavior of the Bi-adsorbed AGNRs, all armchair Al chemisorptions belong to non-magnetic configuration (Table 1 and Figure A2a,b). However, the Al-adsorbed zigzag systems exhibit diverse magnetic behaviors, being different from/similar to the spin arrangements of the Bi-adsorbed ones (Figure A2c,d). The central single-Al adsorption cannot create the magnetic moment and thus preserve the original AFM spin configuration ((the (9)${}_{s}$ case in Table 1). On the other hand, a single Al close to the upper zigzag boundary will partially destroy the spin configuration due to the zigzag edge carbons, depending on the adatom positions, as clearly revealed in Figure A2c under the (1)${}_{s}$ case. The lower zigzag edge preserves the spin-up-dominated configuration (red balls) almost identical to the original case. However, the spin-down arrangement (blue balls) near the upper edge is fully annihilated, and the spin-up distribution might be drastically changed from edge to center. It should have the maximum magnetic moment for the Al adatom near the zigzag edge. On the other hand, one central Al adatom hardly affects the original AFM spin distribution, being similar to the Bi case (Figure 3c). The magnetic properties thoroughly vanish under two Al adatoms near the separate zigzag edges. The above-mentioned features of magnetic properties clearly show the very strong competition/cooperation between the multi-orbital hybridizations of Al-C bonds and the spin states due to zigzag edge carbons in the total ground state energy.

There are certain important differences in energy structures between the Ni-adsorbed systems and the Ti/Fe/Co ones. As for Co adatoms on AGNR, they dominate the low-lying energy bands and present similar spin-split spacing at low energy as Ti and Fe adatoms (Figure A4a,c) for both armchair and zigzag GNRs. However, the Ni adatoms mostly contribute to the valence band −2 eV $\leq \;E^{v}\leq \;0$ eV. It should be noticed that Ni-adsorbed AGNRs possess no magnetic moment (Table 1), resulting in the absence of band split in low energy (Figure A4b). For the zigzag system ones, they exhibit smaller spin splits in low-lying bands compared to others (Figure A4d), owing to the weak magnetic moments.

Although the effects of substrates on the local magnetic moments are not investigated in this work, this is also interesting to briefly mention. For metal substrates, they can be classified into two groups: s-dominated states such as Ag and Au [52,57]; and the p-/d-dominated states such as Al and Ir [58]. With external applied bias voltage, it is reported that the magnetic moments are tunable in the former cases, but less effective for the latter cases. In general, the magnetic moments at the zigzag GNR edges can be destroyed as a result of the interaction between the ribbon edges and the substrate, except the case in which this interaction is sufficiently weak, e.g., H-terminated GNRs on Au(111) [59]. For non-metal substrates, e.g., topological insulator Sb${}_{2}$Te${}_{3}$, H-terminated [60] and H-free graphene nanoribbon (GNR) edges [61], which respectively bind weakly and strongly to the substrate, will exhibit different characteristics. There is induced noncollinear edge magnetism in GNRs in the latter system due to the strong spin-orbit coupling (SOC) of the substrate. In general, any substrate with large SOC could lead to such a phenomenon, unless chemical interactions at the edge destabilize the edge magnetism, for instance heavy metal substrates [51,57,58]. Besides, it is worth knowing that deformation might also affect the magnetism of GNRs. Sinusoidal shear-strained deformations are reported to induce local magnetic moments along the axis of ZGNRs, while uniform shear or axial deformations do not produce magnetization owning to symmetry restrictions [62].

## References

[1] Way A., Murray E., Jacobberger R.M., Saraswat V., Goeltl F., Mavrikakis M., Arnold M.S. (2019). Tightly Pitched sub-10 nm Graphene Nanoribbon Arrays via Seed Mediated Growth on Ge (001). ECS Trans..

[2] Jiao L., Wang X., Diankov G., Wang H., Dai H. (2010). Facile synthesis of high-quality graphene nanoribbons. Nat. Nanotechnol..

[3] Jacobse P.H., Kimouche A., Gebraad T., Ervasti M., Thijssen J., Liljeroth P., Swart I. (2017). Electronic components embedded in a single graphene nanoribbon. Nat. Commun..

[4] Owens F.J. (2008). Electronic and magnetic properties of armchair and zigzag graphene nanoribbons. J. Chem. Phys..

[5] Singh S., Kaur I. (2020). Bandgap engineering in armchair graphene nanoribbon of zigzag-armchair-zigzag based Nano-FET: A DFT investigation. Phys. E Low-Dimens. Syst. Nanostruct..

[6] Han M.Y., Özyilmaz B., Zhang Y., Kim P. (2007). Energy band-gap engineering of graphene nanoribbons. Phys. Rev. Lett..

[7] Bai J., Duan X., Huang Y. (2009). Rational fabrication of graphene nanoribbons using a nanowire etch mask. Nano Lett..

[8] Wang X., Ouyang Y., Li X., Wang H., Guo J., Dai H. (2008). Room-temperature all-semiconducting sub-10-nm graphene nanoribbon field-effect transistors. Phys. Rev. Lett..

[9] Fujii S., Enoki T. (2010). Cutting of oxidized graphene into nanosized pieces. J. Am. Chem. Soc..

[10] Sprinkle M., Ruan M., Hu Y., Hankinson J., Rubio-Roy M., Zhang B., Wu X., Berger C., De Heer W.A. (2010). Scalable templated growth of graphene nanoribbons on SiC. Nat. Nanotechnol..

[11] Jiao L., Xie L., Dai H. (2012). Densely aligned graphene nanoribbons at 35 nm pitch. Nano Res..

[12] Li X., Wang X., Zhang L., Lee S., Dai H. (2008). Chemically derived, ultrasmooth graphene nanoribbon semiconductors. Science.

[13] Lin J., Raji A.R.O., Nan K., Peng Z., Yan Z., Samuel E.L., Natelson D., Tour J.M. (2014). Iron oxide nanoparticle and graphene nanoribbon composite as an anode material for high-performance Li-ion batteries. Adv. Funct. Mater..

[14] Li Y.S., Ao X., Liao J.L., Jiang J., Wang C., Chiang W.H. (2017). Sub-10-nm graphene nanoribbons with tunable surface functionalities for lithium-ion batteries. Electrochim. Acta.

[15] Zou X., Wang L., Yakobson B.I. (2018). Mechanisms of the oxygen reduction reaction on B-and/or N-doped carbon nanomaterials with curvature and edge effects. Nanoscale.

[16] Wang C., Gao H., Li H., Zhang Y., Huang B., Zhao J., Zhu Y., Yuan W.Z., Zhang Y. (2014). Graphene nanoribbons hybridized carbon nanofibers: Remarkably enhanced graphitization and conductivity, and excellent performance as support material for fuel cell catalysts. Nanoscale.

[17] Chang S.L., Wu B.R., Yang P.H., Lin M.F. (2012). Curvature effects on electronic properties of armchair graphene nanoribbons without passivation. Phys. Chem. Chem. Phys..

[18] Wang Y., Zhan H., Yang C., Xiang Y., Zhang Y. (2015). Formation of carbon nanoscrolls from graphene nanoribbons: A molecular dynamics study. Comput. Mater. Sci..

[19] Kawai S., Saito S., Osumi S., Yamaguchi S., Foster A.S., Spijker P., Meyer E. (2015). Atomically controlled substitutional boron-doping of graphene nanoribbons. Nat. Commun..

[20] Nguyen D.K., Tran N.T.T., Nguyen T.T., Lin M.F. (2018). Diverse electronic and magnetic properties of chlorination-related graphene nanoribbons. Sci. Rep..

[21] Huang Y., Chang C., Lin M.F. (2007). Magnetic and quantum confinement effects on electronic and optical properties of graphene ribbons. Nanotechnology.

[22] Son Y.W., Cohen M.L., Louie S.G. (2006). Half-metallic graphene nanoribbons. Nature.

[23] Rigo V., Martins T., da Silva A.J., Fazzio A., Miwa R. (2009). Electronic, structural, and transport properties of Ni-doped graphene nanoribbons. Phys. Rev. B.

[24] Yu S.S., Zheng W.T., Jiang Q. (2009). Electronic properties of nitrogen-atom-adsorbed graphene nanoribbons with armchair edges. IEEE Trans. Nanotechnol..

[25] Srivastava P., Dhar S., Jaiswal N.K. (2015). Potential spin-polarized transport in gold-doped armchair graphene nanoribbons. Phys. Lett. A.

[26] Johnson J.L., Behnam A., Pearton S., Ural A. (2010). Hydrogen sensing using Pd-functionalized multi-layer graphene nanoribbon networks. Adv. Mater..

[27] Lin J., Peng Z., Xiang C., Ruan G., Yan Z., Natelson D., Tour J.M. (2013). Graphene nanoribbon and nanostructured SnO2 composite anodes for lithium ion batteries. ACS Nano.

[28] Lin M.C., Gong M., Lu B., Wu Y., Wang D.Y., Guan M., Angell M., Chen C., Yang J., Hwang B.J. (2015). An ultrafast rechargeable aluminium-ion battery. Nature.

[29] Li Y., Trujillo M.A., Fu E., Patterson B., Fei L., Xu Y., Deng S., Smirnov S., Luo H. (2013). Bismuth oxide: A new lithium-ion battery anode. J. Mater. Chem. A.

[30] Wang Z., Xiao J., Li M. (2013). Adsorption of transition metal atoms (Co and Ni) on zigzag graphene nanoribbon. Appl. Phys. A.

[31] Sevinçli H., Topsakal M., Durgun E., Ciraci S. (2008). Electronic and magnetic properties of 3 d transition-metal atom adsorbed graphene and graphene nanoribbons. Phys. Rev. B.

[32] Kresse G., Furthmüller J. (1996). Efficient iterative schemes for ab initio total-energy calculations using a plane-wave basis set. Phys. Rev. B.

[33] Kresse G., Joubert D. (1999). From ultrasoft pseudopotentials to the projector augmented-wave method. Phys. Rev. B.

[34] Blöchl P.E. (1994). Projector augmented-wave method. Phys. Rev. B.

[35] Perdew J.P., Burke K., Ernzerhof M. (1996). Generalized gradient approximation made simple. Phys. Rev. Lett..

[36] Lin S.Y., Lin Y.T., Tran N.T.T., Su W.P., Lin M.F. (2017). Feature-rich electronic properties of aluminum-adsorbed graphenes. Carbon.

[37] Lebon A., Carrete J., Longo R., Vega A., Gallego L. (2013). Molecular hydrogen uptake by zigzag graphene nanoribbons doped with early 3d transition-metal atoms. Int. J. Hydrogen Energy.

[38] Cocchi C., Prezzi D., Calzolari A., Molinari E. (2010). Spin-transport selectivity upon Co adsorption on antiferromagnetic graphene nanoribbons. J. Chem. Phys..

[39] Ohta T., Bostwick A., McChesney J.L., Seyller T., Horn K., Rotenberg E. (2007). Interlayer interaction and electronic screening in multilayer graphene investigated with angle-resolved photoemission spectroscopy. Phys. Rev. Lett..

[40] Ruffieux P., Cai J., Plumb N.C., Patthey L., Prezzi D., Ferretti A., Molinari E., Feng X., Mullen K., Pignedoli C.A. (2012). Electronic structure of atomically precise graphene nanoribbons. Acs Nano.

[41] Senkovskiy B.V., Fedorov A.V., Haberer D., Farjam M., Simonov K.A., Preobrajenski A.B., Mårtensson N., Atodiresei N., Caciuc V., Blügel S. (2017). Semiconductor-to-Metal Transition and Quasiparticle Renormalization in Doped Graphene Nanoribbons. Adv. Electron. Mater..

[42] Chen J.W., Huang H.C., Convertino D., Coletti C., Chang L.Y., Shiu H.W., Cheng C.M., Lin M.F., Heun S., Chien F.S.S. (2016). Efficient n-type doping in epitaxial graphene through strong lateral orbital hybridization of Ti adsorbate. Carbon.

[43] Virojanadara C., Watcharinyanon S., Zakharov A., Johansson L.I. (2010). Epitaxial graphene on 6 H-SiC and Li intercalation. Phys. Rev. B.

[44] Söde H., Talirz L., Gröning O., Pignedoli C.A., Berger R., Feng X., Müllen K., Fasel R., Ruffieux P. (2015). Electronic band dispersion of graphene nanoribbons via Fourier-transformed scanning tunneling spectroscopy. Phys. Rev. B.

[45] Chen Y.C., De Oteyza D.G., Pedramrazi Z., Chen C., Fischer F.R., Crommie M.F. (2013). Tuning the band gap of graphene nanoribbons synthesized from molecular precursors. ACS Nano.

[46] Huang H., Wei D., Sun J., Wong S.L., Feng Y.P., Neto A.C., Wee A.T.S. (2012). Spatially resolved electronic structures of atomically precise armchair graphene nanoribbons. Sci. Rep..

[47] Chen H.H., Su S., Chang S.L., Cheng B.Y., Chen S., Chen H.Y., Lin M.F., Huang J. (2015). Tailoring low-dimensional structures of bismuth on monolayer epitaxial graphene. Sci. Rep..

[48] Tapasztó L., Dobrik G., Lambin P., Biro L.P. (2008). Tailoring the atomic structure of graphene nanoribbons by scanning tunnelling microscope lithography. Nat. Nanotechnol..

[49] Liao Z., Sandonas L.M., Zhang T., Gall M., Dianat A., Gutierrez R., Mühle U., Gluch J., Jordan R., Cuniberti G. (2017). In-situ stretching patterned graphene nanoribbons in the transmission electron microscope. Sci. Rep..

[50] Rezapour M.R., Lee G., Kim K.S. (2020). A high performance N-doped graphene nanoribbon based spintronic device applicable with a wide range of adatoms. Nanoscale Adv..

[51] Roche S., Åkerman J., Beschoten B., Charlier J.C., Chshiev M., Dash S.P., Dlubak B., Fabian J., Fert A., Guimarães M. (2015). Graphene spintronics: The European Flagship perspective. 2D Mater..

[52] Ganguly S., Basu S. (2017). Adatoms in graphene nanoribbons: Spintronic properties and the quantum spin Hall phase. Mater. Res. Express.

[53] Liu J., Li C., Jin W., Lefkidis G., Hübner W. (2021). Long-Distance Ultrafast Spin Transfer over a Zigzag Carbon Chain Structure. Phys. Rev. Lett..

[54] Liu H., Kondo H., Ohno T. (2016). Spintronic transport in armchair graphene nanoribbon with ferromagnetic electrodes: Half-metallic properties. Nanoscale Res. Lett..

[55] Tanaka M. (2020). Recent progress in ferromagnetic semiconductors and spintronics devices. Jpn. J. Appl. Phys..

[56] Han W., Kawakami R.K., Gmitra M., Fabian J. (2014). Graphene spintronics. Nat. Nanotechnol..

[57] Li Y., Zhang W., Morgenstern M., Mazzarello R. (2013). Electronic and magnetic properties of zigzag graphene nanoribbons on the (111) surface of Cu, Ag, and Au. Phys. Rev. Lett..

[58] Li Y., Subramaniam D., Atodiresei N., Lazić P., Caciuc V., Pauly C., Georgi A., Busse C., Liebmann M., Bluegel S. (2013). Absence of edge states in covalently bonded zigzag edges of graphene on Ir (111). Adv. Mater..

[59] Chen J., Vanin M., Hu Y., Guo H. (2012). Tuning the magnetic moments in zigzag graphene nanoribbons: Effects of metal substrates. Phys. Rev. B.

[60] Zhang W., Hajiheidari F., Li Y., Mazzarello R. (2016). Electronic and magnetic properties of H-terminated graphene nanoribbons deposited on the topological insulator Sb 2 Te 3. Sci. Rep..

[61] Zhang W., Hajiheidari F., Mazzarello R. (2017). Chiral magnetic interactions in graphene nanoribbons on topological insulator substrates. Phys. Rev. B.

[62] Al-Aqtash N.M., Sabirianov R.F. (2014). Spin density waves in periodically strained graphene nanoribbons. Nanoscale.

